# Glioblastoma multiform with ipsilateral carotid artery stenosis: carotid artery stent promote tumor growth?

**DOI:** 10.1186/s12957-016-0782-z

**Published:** 2016-02-06

**Authors:** Ziqi Xu, Benyan Luo, Qidong Wang, Zhiyi Peng, Hui Liang

**Affiliations:** 1 Department of Neurology, The First Affiliated Hospital of the College of Medicine, Zhejiang University, Hangzhou, China; 2 Department of Radiology, The First Affiliated Hospital of the College of Medicine, Zhejiang University, No. 79 Qingchun Road, Hangzhou, 310003 China

**Keywords:** Carotid artery stenosis, Stent, Ischemic stroke, Glioblastoma, Misdiagnosis

## Abstract

**Background:**

Ischemic stroke and glioblastoma multiforme have similar features on anatomic magnetic resonance imaging (MRI) and thus may require a surgical biopsy for a definitive diagnosis.

**Case presentation:**

A 55-year-old male complained of dysphasia for 4 weeks and continuous deterioration for 5 days. Cerebral infarction was considered based on MRI, which showed hyperintensity at the border zone of the left hemisphere, and computed tomography angiography (CTA) showed left carotid artery severe stenosis. The patient underwent placement of a left carotid artery stent, and his symptoms recurred 2 months after carotid artery stent (CAS). MRI showed multiple ring-enhanced lesions in the left temporal, parietal, and occipital lobes accompanied by massive brain edema. The final pathologic diagnosis was glioblastoma multiforme.

**Conclusion:**

Although there is no evidence that stent therapy for carotid artery stenosis will worsen an ipsilateral glioblastoma, we should be careful to perform surgeries involving carotid artery stents when the patient has a glioblastoma.

## Background

Ischemic stroke is a leading health problem in China, and is the most common disease in elderly people [1]. Glioblastoma multiforme (GBM) is a World Health Organization (WHO) grade IV tumor with a dismal prognosis; <10 % of patients are alive after 2 years [2]. Co-existing ischemic stroke with carotid artery stenosis and GBM is rare. The patient described herein presented three times with ischemic strokes complicated by left carotid artery severe stenosis. Magnetic resonance imaging (MRI) showed an infarct pattern consistent with a watershed infarction. Two months after left carotid artery stent (CAS), multiple lesions were noted in the left cerebral hemisphere with massive edema, and a GBM was diagnosed post-operatively. There are some issues worthy of discussion about the clinical diagnosis, therapy, and blood flow after CAS-induced deterioration.

## Case presentation

**Fig. 1:**
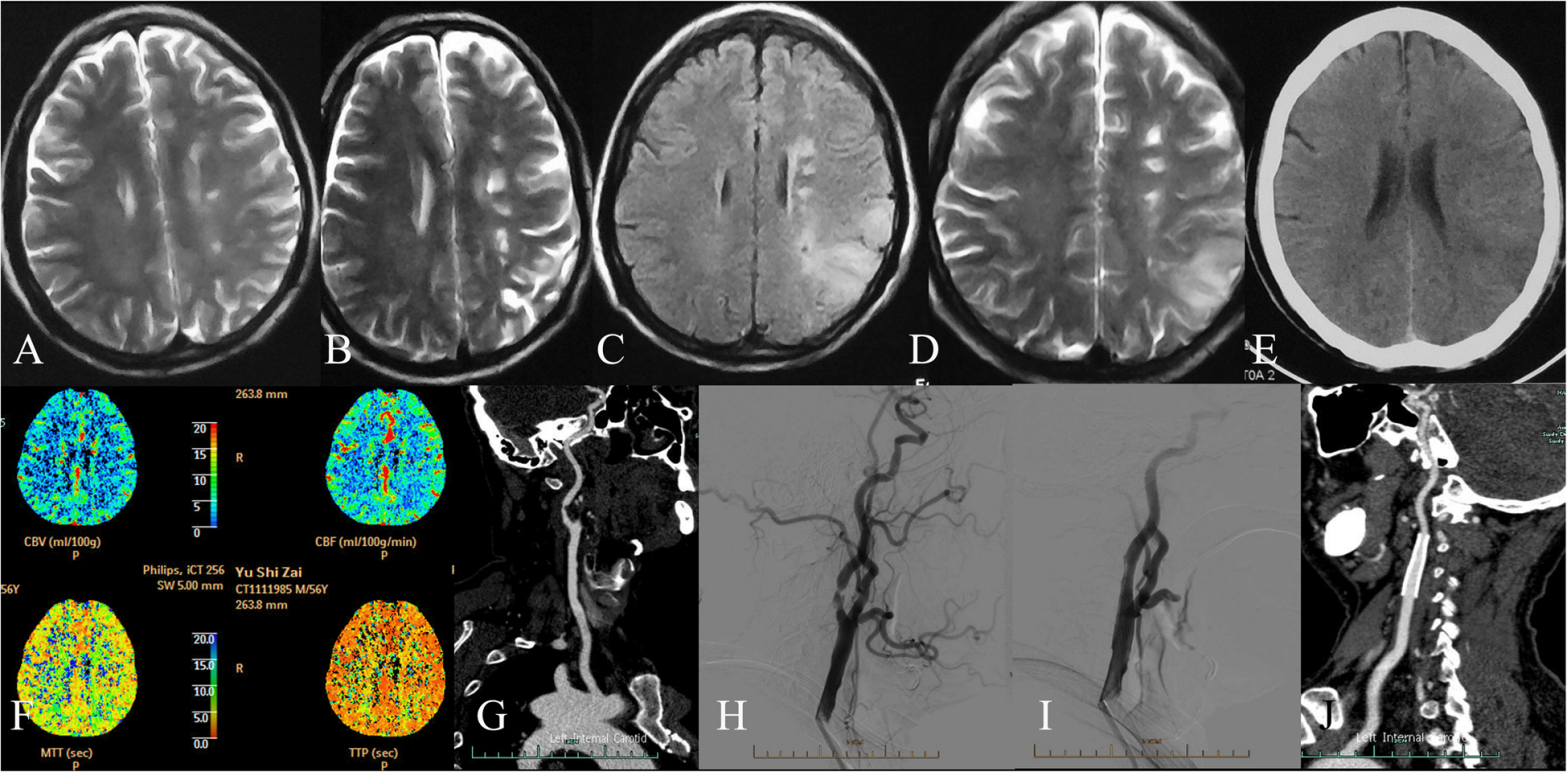
Patient images before carotid artery stenting. **a** (T2WI [2013-5-5]) and **b** (T2WI [2013-10-14]):T2-weighted image shows internal border zone cerebral infarction. **c**, **d** (2014-12-2): T2 flair image and T2-weighted image showed hyperintensity in the left temporal and occipital lobes. **e** (2014-12-26): CT image showed low density in the left internal border zone and temporal and occipital lobes. **f** CT perfusion image showed mild cerebral ischemia of left cerebral hemisphere. **g** CT angiography (2015-01-03) showed severe left carotid artery stenosis. **h**, **i** DSA showed severe left carotid artery stenosis with stenting therapy. (**j**): 3-month follow-up CT angiography of left carotid artery after carotid artery stenting

**Fig. 2:**
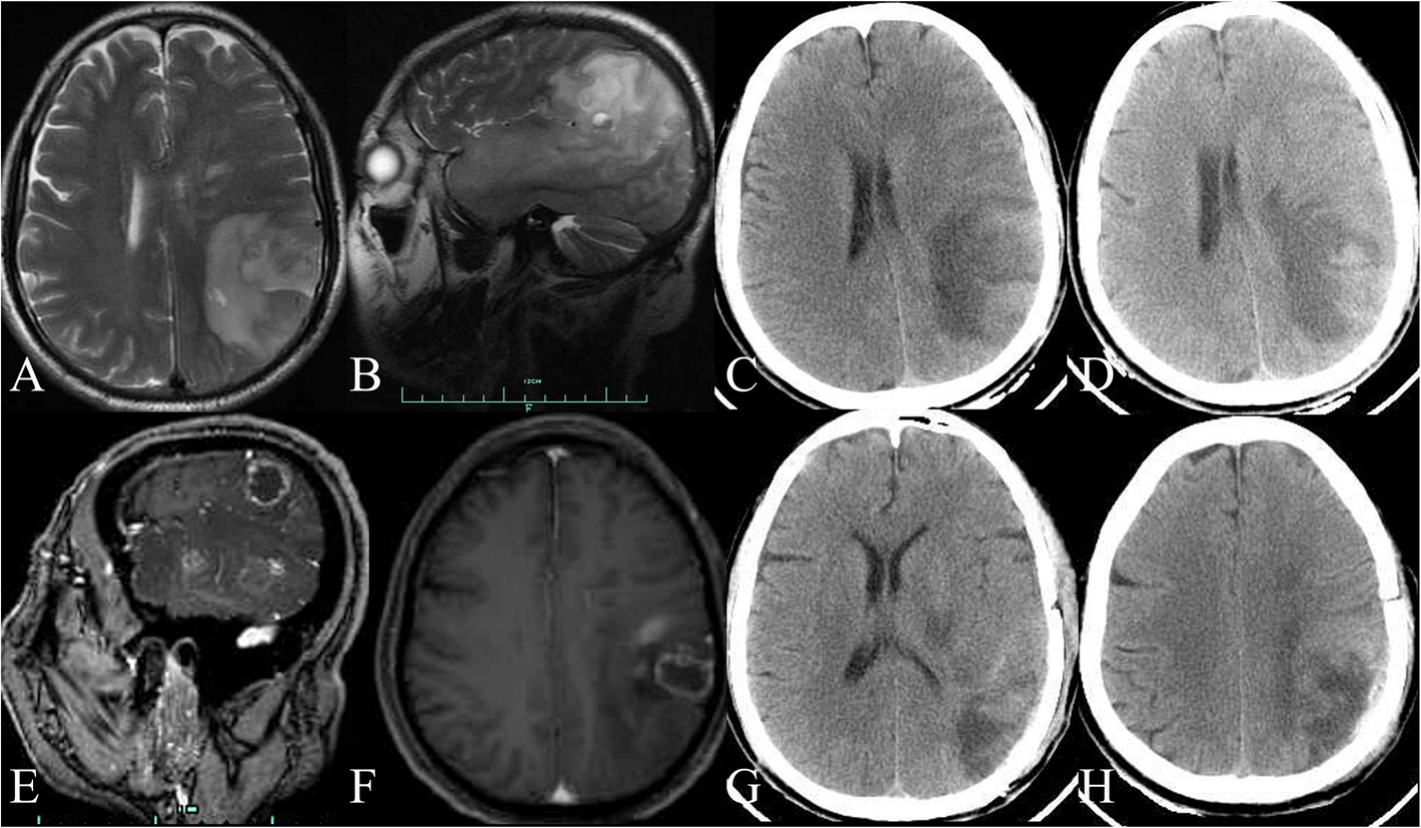
Images 2 months after carotid artery stenting. **a**, **b** (2015-3-24): T2-weighted image showed a massive lesion with edema in the left temporal and occipital lobes. **c** (2015-3-24): CT image also found a massive lesion with edema in the left temporal and occipital lobes. **d** (2015-3-29): The repeat cranial CT revealed that cerebral edema worsened. **e**, **f** (2015-4-11): contrast T1-weighted images showed multiple ring-enhanced lesions with massive edema in the left temporal and occipital lobes. **g**, **h** (2015-4-29): Repeat cranial CT scans showed edema regressed 2 weeks post-operatively

A 55-year-old male patient complained of dysphasia for 4 weeks and continuous deterioration for 5 days. The patient had a history of ischemic stroke (Fig. 1a, b), which manifested as right limb numbness and amaurosis 1 year ago. He had a history of hypertension for 10 years, a myocardial infarction 3 years ago, and cigarette smoking for 40 years, but no history of diabetes, hyperlipidemia, and alcohol consumption. The patient exhibited a disorder of linguistic expression and long-term memory impairment without a limb motor disorder. Cranial MRI confirmed an ischemic stroke at a local hospital (Fig. 1c, d). The symptoms were relieved after medical treatment. The dysphasia relapsed 5 days before admission. Cranial computed tomography (CT) showed a hypodense lesion in the left temporal and occipital lobes (Fig. 1e). Cerebral infarction was considered based on serial cranial MRI, which showed hyperintensity at the internal border zone and the border zone between the left temporal and occipital lobes, and Doppler ultrasound revealed severe left carotid artery stenosis. The patient was transferred to our stroke center for further treatment. At the time of admission, the vital signs were normal. A neurologic examination showed mild dysphasia, normal cranial nerves, normal limb motor function, and negative Babinski signs bilaterally. CT perfusion imaging showed mild ischemic in the territory of the left middle cerebral artery (Fig. 1f). Cervical computed tomography angiography (CTA) confirmed the first segment of the carotid artery with severe stenosis (Fig. 1g). An electrocardiogram revealed an abnormal Q-wave involving the inferior wall, which was consistent with an inferior myocardial infarction. Homocysteine (35.3 μmol/L) and the following laboratory investigations were normal: triglycerides, total cholesterol, low-density lipoprotein cholesterol, antinuclear antibodies, anti-neutrophil cytoplasmic antibodies, prothrombin time, and tumor markers. The patient was given medical treatment consisting of aspirin (100 mg per day), clopidogrel (75 mg), and atorvastatin (20 mg). The patient received combined anti-platelet treatment with 100 mg of aspirin and 75 mg of clopidogrel daily for a week prior to the intervention. Under local anesthesia, the patient underwent placement of a left carotid artery stent (Fig. 1h, i). The patient was discharged 3 days post-operatively. Two months after CAS, the patient experienced right limb numbness and mild dysphasia with a severe headache. The recurrent symptoms and each episode lasted approximately 2 min. An emergency cranial MRI showed massive hyperintense lesions in the left temporal and occipital lobes with ventricular compression (Fig. 2a, b). The cranial CT performed on the same day indicated no cerebral hemorrhage (Fig. 2c). A neurologic examination was also negative. On the basis of a detailed medical history, a diagnosis of hyperfusion was proposed; an anti-hypertensive and mannitol were introduced, and aspirin was discontinued. Despite the medical treatment, the headache worsened. A repeat cranial CT revealed that the cerebral edema had worsened (Fig. 2d). Based on the failed medical treatment and imaging features, a brain tumor was considered. Repeat MRI showed multiple ring-enhanced lesions in the left temporal, parietal, and occipital lobes complicated by massive brain edema (Fig. 2e, f). The patient underwent whole-body positron emission tomography–computed tomography (PET/CT), which showed that the increased FDG metabolism in the left parietal and temporal lobes was consistent with malignant lesions. Revised medical treatment included albumin, furosemide, and dexamethasone. Then, the patient was transferred to the neurosurgery ward pre-operatively. The final pathologic diagnosis was glioblastoma multiforme (WHO grade IV). A post-operative intracranial CT showed regression of the cerebral edema (Fig. 2g, h).

### Discussion

GBM is a malignant tumor with a dismal prognosis that is often associated with extensive angiogenesis due to tumor secretion and local effects exerted by vascular endothelial growth factor, a major regulator of angiogenesis and other cytokines [3]. The patient described herein initially presented with ischemic stroke and severe ipsilateral carotid artery stenosis and an intracranial massive lesion worsened after CAS. Cerebral hyperfusion syndrome was suspected due to headaches, cerebral edema, focal neurologic deficits, serial images, and a pathologic diagnosis of a GBM. Hyperperfusion syndrome is a relatively rare complication of carotid artery revascularization procedures and may have adverse clinical consequences in 1.1–25.0 % of patients after CAS [4]. Delayed hyperperfusion syndrome has been reported [4], which may be related to prolonged impairment of cerebrovascular autoregulation. Some questions remain which should be addressed. The course of the disease was approximately 2 years, and the patient had numerous atherosclerotic risks, including hypertension, smoking, and coronary artery disease. The patient had right limb weakness and numbness, amaurosis, and mild dysphasia. MRI showed a left internal border zone cerebral infarction and severe left carotid artery stenosis. Thus, an ischemic stroke was not only misdiagnosed, but CAS was reasonable. Imaging features of GBM have no special characteristics. Multiple model MRI images are useful in the diagnosis of GBM and its tumor grade [2, 5–7]. The characteristic MRI findings of GBM include enhanced heterogeneous ring mass lesions with significant peritumoral cerebral edema, necrosis, or hemorrhage [5, 7]. These features also occur in patients with ischemic strokes [8, 9]. GBMs often pose a diagnostic dilemma on anatomic MRI and may require a surgical biopsy for a definitive diagnosis. In the current patient, whole-body PET/CT revealed no systemic tumor and brain metastasis was ruled out.

Did CAS prompt the growth of the GBM? The left cerebral hemisphere was ischemic secondary to severe carotid artery stenosis, while the GBM was dependent on the blood supply. Brain ischemia and the GBM were improved when the left carotid artery stenosis was recanalized, which may induce tumor growth. Relative cerebral blood volume (rCBV) maps and measurements have been shown to correlate reliably with tumor grade and histologic findings of increased tumor vascularity [5, 7, 10]. In addition, a study has proved that if the rCBV is >1.75, there is a high probability that the tumor will be a high-grade glioma [5]. GBM complicated with carotid artery stenosis is rarely reported, and there is insufficient evidence to prove a relationship between a worsening tumor and CAS.

## Conclusions

A GBM is a high-grade tumor with a dismal prognosis; complication with severe ipsilateral carotid artery stenosis is rare. Although ischemic stroke is a common disease, we should make the diagnosis cautiously to avoid misdiagnosis. There is insufficient evidence that stent therapy for carotid artery stenosis will aggravate an ipsilateral GBM. Thus, we should be careful in performing CAS when a patient has a concomitant GBM.

### Consent

Written informed consent was obtained from the patient for publication of this case report and any accompanying images. A copy of the written consent is available for review by the Editor-in-Chief of this journal.
